# New Numerical Score and Stepwise Algorithm for the Classification and Management of Laryngomalacia

**DOI:** 10.1055/s-0044-1801317

**Published:** 2025-05-29

**Authors:** Ahmed Elsobki, Hemmat Baz, Reham AE Ibrahim, Menna Ibrahim Hashish, Mohamed E. El-Deeb, Noha Ahmed El-Kholy

**Affiliations:** 1 Department of Otorhinolaryngology, Head and Neck Surgery, Faculty of Medicine, Mansoura University, Mansoura, Egypt; 2 Department of Phoniatrics, Faculty of Medicine, Mansoura University, Mansoura, Egypt; 3 Department of Phoniatrics, Faculty of Medicine, Assuit University, Assuit, Egypt; 4 Department of Pediatrics, Faculty of Medicine, Mansoura University, Mansoura, Egypt; 5 Department of Otorhinolaryngology, Faculty of Medicine, Kafrelsheikh University, Kafr El-Sheikh, Egypt

**Keywords:** laryngomalacia, scoring system, algorithm, larynx

## Abstract

**Introduction** The classification and management of laryngomalacia are challenging topics that are continuously updated and modified by pediatric airway surgeons. However, a numerical stratification of the patients to decide on the conservative management or intervention has not yet been established.

**Objective** To provide an easy approach to cases of laryngomalacia by adopting an updated scoring system with a stepwise management algorithm.

**Methods** We conducted a prospective study that included patients diagnosed with laryngomalacia over three years. The proposed symptom and history score was used to categorize patients into mild, moderate, and severe grades. Then, the examination and investigation scores designed were applied to the selected cases according to the management algorithm. Basic data from the patients and their flow throughout the study were assessed.

**Results** The study included 112 patients with a mean age of 4.3 ± 2.2 months. In total, 44 (39.3%) cases were considered mild, 48 (42.86%), moderate, and 20 (17.85%), severe. The examination score was used to assess 68 out of 112 patients, including all moderate and severe cases of laryngomalacia. All of the mild cases were followed up, and none required surgery. The investigation score was applied to 55 cases, including all the severe cases, as preoperative evaluation, and 35 out of 48 moderate cases.

**Conclusion** This newly proposed scoring system with the associated algorithm is an easily applicable way to deal with, classify, and properly manage laryngomalacia cases.

## Introduction

Laryngomalacia is the most prevalent congenital laryngeal condition, and it is characterized by dynamic prolapse of redundant supraglottic structures, causing inspiratory stridor. It accounts for about 75% of the causes of stridor in infants. 1 2

Over the past decades, there has been an ongoing debate on the grades and stages of laryngomalacia and how to classify the patients and manage the condition. 3 4 5 Many aspects of laryngomalacia can affect the surgeon's decision, and many argue 2 about the strict definition of mild, moderate, and severe cases and how to proceed from diagnosis to either the conservative approach or intervention. Moreover, most papers 48 in this field suffer from a lack of a definitive set of indications for supraglottoplasty, use, and abuse of possible investigations in a scaling fashion.

There is an increasing need to stratify laryngomalacia patients so that physicians can make an easier and proper management decision. Thus, various classifications have been described by authors 2 5 6 based on history questionnaires and the severity of the collapse of the supraglottic structures.

In 2016, the International Pediatric Otolaryngology Group (IPOG) published their laryngomalacia consensus recommendations, 3 with a classification dividing the cases into mild, moderate, and severe grades. However, a numerical score to globally assess laryngomalacia patients is currently lacking, especially for the moderate and moderately severe cases.

The current paper has the goal of aiding in the assessment of and decision-making regarding laryngomalacia through a proposed numerical score and a stepwise algorithm. To the best of our knowledge, the present is the first report of a proposed algorithm for the management of laryngomalacia based on a numerical scoring system.

## Methods

We conducted a prospective study involving all patients diagnosed with laryngomalacia at our tertiary referral center throughout 3 years. The study was approved by the institutional Ethical Review Board (R.22.02.1627), and written informed consent was obtained from the parents of the patients included.

### Subjects

**Fig. 1:**
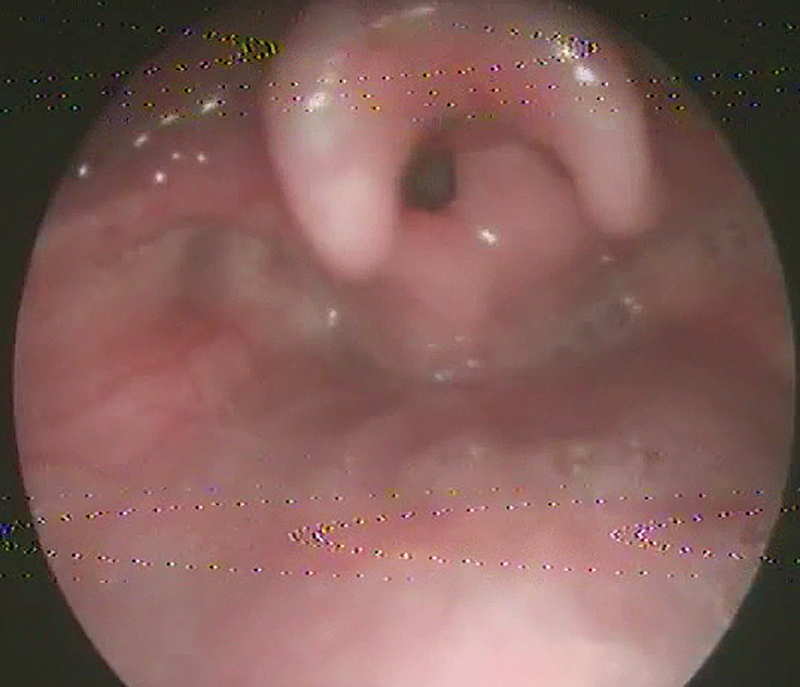
Type I laryngomalacia (redundant arytenoid mucosa).

The patients were enrolled in the protocol using strict inclusion and exclusion criteria from September 2018 to August 2021. The inclusion criteria were pediatric patients with a diagnosis of laryngomalacia documented by awake fiberoptic laryngoscopic examination in the outpatient clinic ( Figs. 1 2 3 ). The exclusion criteria were patients with adult-onset laryngomalacia and supraglottic (arytenoid) collapse complicating posterior glottis expansion surgery.

**Fig. 2:**
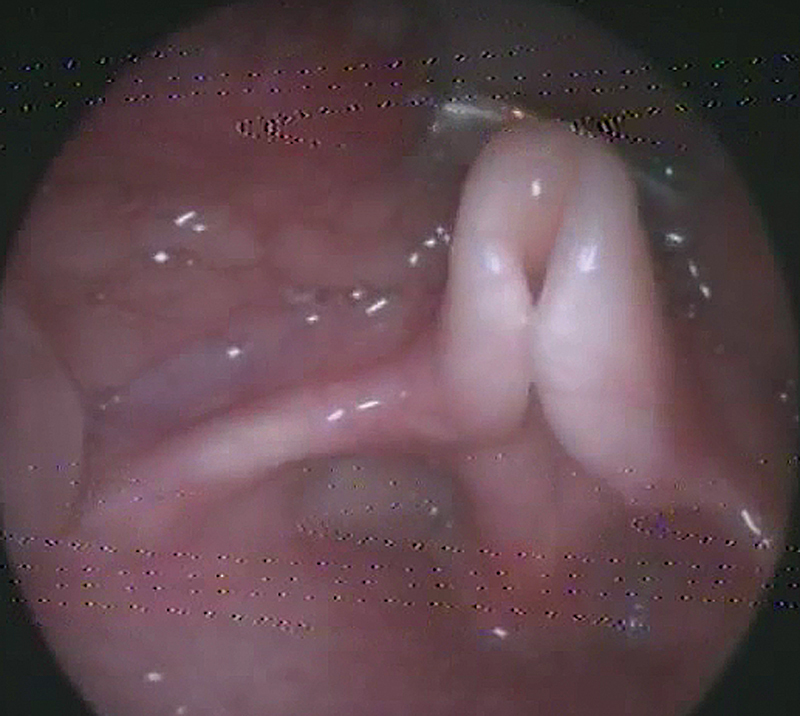
Type-II laryngomalacia (short aryepiglottic fold with curled epiglottis).

**Fig. 3:**
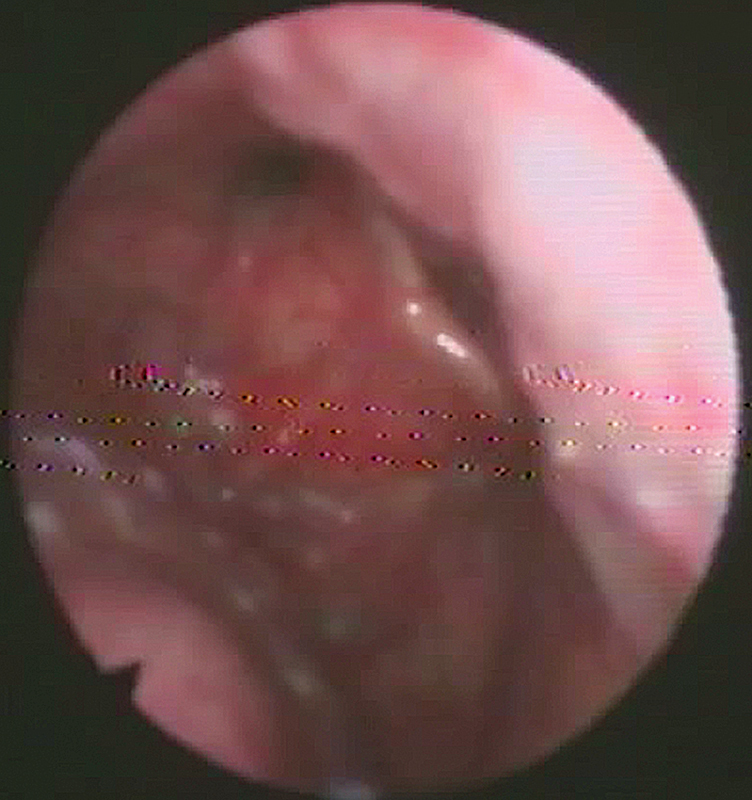
Type-III laryngomalacia (epiglottic collapse).

The scoring system was divided into three parts: the history and symptom score, the examination score, and the investigation score. Each of them was the sum of a certain number of checkpoints, and each checkpoint was categorized into 0, 1, 2, or 3 according to the severity, and 3 meant the most severe. The sum of these points represented the score ( Table 1 ). The three parts of the scoring system are explained as follows:

**Table 1:** Proposed laryngomalacia scoring system

	0	1	2	3
**1. History and symptom score (ranges from 0–15 points)**				
▪Inspiratory sound	Intermittent	Persistent, mild (not associated with sucked in chest skin)	Persistent, severe (associated with sucked-in chest skin)	
▪Cyanotic spells	Never			Present
▪Interrupted sleep	Never	Infrequent (not in every sleep)	Frequent (persistent in every sleep)	
▪Is the baby growing?	Yes	Less than ideally	Losing weight	
▪Choking/coughing during feeding	Never	Infrequent (not in every feeding)	Frequent (persistent in every feeding)	
▪Regurgitation/vomiting during/ after feeding	Never	Infrequent (not in every feeding)	Frequent (Persistent in every feeding)	
▪History of hospital/ICU admission due to respiratory compromise	Never	Once	More than once	
**2. Examination score (ranges from 0–13 points)**				
▪Growth chart	Normal	Moderate delay	Marked delay	
▪Chest retractions	Absent	Suprasternal	Intercostal or subcostal	Pectus excavatum
▪Degree of collapse	25–50% of vocal cords obscured	75% of vocal cords obscured	100% of vocal cords obscured	
▪Swallowing assessment				
➢ FEES	Normal	Penetration	Aspiration	
▪Developmental assessment	Normal	Mild impairment	Global delay	
▪Associated congenital anomalies	None	One	More than one	
**3. Investigation score (ranges from 0–10 points)**				
▪ECHO	normal	Associated congenital anomaly	Mild or moderate pulmonary hypertension	Severe pulmonary hypertension or right-sided heart failure
▪Overnight pulse oximetry	No nocturnal desaturation	O _2_ desaturation nadir: 86–91%	O _2_ desaturation nadir 76–85%	< 75%
▪Synchronous airway lesion	No other lesions	One lesion	More than one lesion	
▪GERD assessment				
➢ Modified barium swallow	Normal	Penetration	Aspiration	
➢ pH meter	RI < 3%	RI: 3–7%	RI > 7%	

**Abbreviations:** ECHO, echocardiography; FEES, functional endoscopic evaluation of swallowing; GERD, gastroesophageal reflux disease; ICU, intensive care unit; RI, reflux index.

### History and Symptom Score

It consisted of seven questions or symptoms, asked to the caregiver ( Table 1 ). Accordingly, each symptom was assigned a number (0, 1, 2, or 3) according to its severity. The sum of these numbers represented the history and symptom score, which ranged from 0 to 15.

### Examination Score

The six items assessed in the examination score included: growth, chest retractions, degree of supraglottic collapse, swallowing evaluation, development, and if there were any associated congenital anomalies. The total examination score was 13.

#### Growth Chart

The World Health Organization's (WHO) weight for length growth charts, from birth to 2 years, were used to assess the patients' growth, 7 where normal patients had a Z score (−2 to +2), moderate delay patients; underweight and stunted, had a Z score between - 2 and – 3 and severe delay cases; severe underweight and severe stunted, have a Z score below – 3. The cases of moderate and severe delay represented infants with failure to thrive (FTT).

#### Degree of Collapse

The degree of severity of collapse of the supraglottic structures is graded into three points based on the percentage of the area observed on the vocal cords during the awake flexible fiberoptic examination. It was assessed according to previously-reported scoring of the arytenoid and epiglottic collapse. 5

#### Swallowing Assessment

The affection of swallowing function was graded by fiberoptic endoscopic evaluation of swallowing (FEES). The FEES examinations were performed in the endoscopy room with a consultant phoniatrician. The infants were typically positioned on a caregiver's lap in an upright or semi-setting position. For infants under the age of 1 year, topical anesthesia was not commonly administered. For older infants, a small amount of topical 2% lidocaine was instilled in the nasal cavity. A flexible video bronchoscope with an outer diameter of 3.1 mm (BF-XP190, Olympus Medical, Hamburg, Germany) was advanced through the nasal cavity. The patients were then fed an age-appropriate diet with different consistencies. For most infants, formula or breast milk was given via a bottle. For the FEES studies, the presence of aspiration and penetration in the different consistencies was noted. 8

#### Developmental Assessment

Griffiths-III is the latest version of the Griffiths Mental Development Scale (GMDS), launched in 2016 to replace the previous versions and widen its application to begin from the first month of life. It provides an overall measure of development across five domains: foundations of learning; language and communication; eye and hand coordination; personal-social-emotional; and gross motor assessment. The raw score of each domain was calculated by the examiner to be reviewed in the norms table to developmental age and percentile. These percentiles ranged from 1 to 99, and the infant was considered delayed if the score was below the first percentile. The interpretation of the GMDS-III was divided into: normal – percentiles of all domains within the normal range; mild impairment – one out of five domains below the normal range; and global delay – two or more domains below the normal range. 9 This score was assessed and reported by a senior pediatric consultant.

### Investigation Score

The investigation score ranged from 0 to 10, and it involved echocardiography, overnight pulse oximetry, presence or absence of synchronous airway lesion, and gastroesophageal reflux disease (GERD) assessment. Pulmonary hypertension was classified as mild, moderate, or severe according to previously-reported 10 values: mild – 25 mmHg to 35 mmHg; moderate – 35 mmHg to 45 mmHg; and severe – > 45 mmHg. The lowest oxygen saturation levels overnight were graded according to oxygen nadir levels reported by Katz et al. 11 As a definitive diagnosis of synchronous airway lesions could not be established through awake flexible laryngoscopy, a full dynamic airway evaluation was performed, either as a part of the diagnostic work-up preoperatively, or perioperatively, under total intravenous general anesthesia while the child was spontaneously ventilating. Then, a 30° endoscope was used in conjunction with a Macintosh blade laryngoscope (Luxamed, Germany) to complete the evaluation. The presence or absence of synchronous airway lesions and their number were reported. 12

Various methods were used to assess GERD, including modified barium swallow (MBS) and pH meter. The MBS studies were performed by a phoniatric consultant and a radiologist in the Fluoroscopic Unit at the Radiology Department. The patients were offered various liquid consistencies of barium and/or pureed and solid foods mixed with barium (one teaspoon of barium mixed with 20 mL of milk). The presence and type of aspiration with different textures of food material were recorded using the Penetration-Aspiration Scale (PAS), which ranges from 1 to 8, as follows: 1–material does not enter the airway; 2–material enters the airway, but remains above the vocal folds, and is ejected from the airway; 3–material enters the airway, remains above the vocal folds, and is not ejected from the airway; 4–material enters the airway, contacts the vocal folds, and is ejected from the airway, PAS 5: Material enters the airway, contacts the vocal folds, and is not ejected from the airway; 6 – material enters the airway, passes below the vocal folds, and is ejected into the larynx or out of the airway; 7–material enters the airway, passes below the vocal folds, and is not ejected from the trachea despite effort; and 8–material enters the airway, passes below the vocal folds, and no effort is made to eject (silent aspiration). The patients were categorized according to their normal swallowing function (PAS 1), laryngeal penetration only (PAS 2 to 5), and aspiration (PAS 6 to 8). 13 The maximum radiation exposure was kept to a limit of 2 minutes to stay within the safety margins. Using a 24-hour pH meter, a pH < 4 in the esophagus was generally considered an acid reflux episode, which was then expressed in terms of the reflux index (RI, the percentage of time a pH < 4 was measured). 14

### Algorithm

**Fig. 4:**
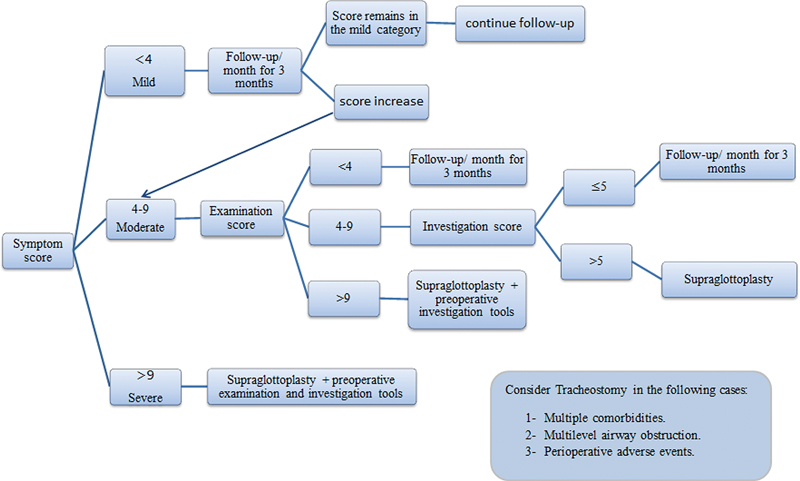
Stepwise algorithm proposed for the management of cases of laryngomalacia.

The scoring process followed an algorithmic stepwise fashion through three stages ( Fig. 4 ).

#### The First Stage

In this stage, the laryngomalacia patients were classified into three grades through the application of the history and symptom score ( Table 1 ): mild – score < 4; moderate – score from 4 to 9; and severe – score > 9. Then, the cases were managed according to their scores. The mild group was conservatively managed and followed up every month for three months until the resolution or progression of the symptoms. The moderate group was managed in the second stage. Patients in the severe group were considered surgical candidates for supraglottoplasty, but they were also managed in the second and third stages to achieve a baseline evaluation before surgery.

#### The Second Stage

This applied to the moderate and severe cases. The moderate cases were divided into three subgroups based on their examination score ( Table 1 ): patients with scores < 4 were managed conservatively; those with scores > 9 were prepared for supraglottoplasty; and subjects with scores from 4 to 9 went on to the third stage of the assessment.

#### The Third Stage

This stage applied to all patients planned for supraglottoplasty as a preoperative evaluation, as well as those with examination scores from 4 to 9. The application of the investigation score to subjects with examination scores from 4 to 9 resulted in their classification into 2 groups: those with investigation scores ≤ 5 were managed by watchful waiting, while those with higher scores were scheduled for supraglottoplasty.

### Acid Suppression Therapy

The IPOG consensus recommendations 3 regarding the step-up and step-down regimens for acid suppression treatment were followed. The step-up regimen started with a single therapy with proton pump inhibitors (PPIs) or histamine type-2 receptor antagonists (H2RAs), which was then stepped-up to dual-acid suppression if the symptoms were not controlled. In the step-down regimen, therapy started with both agents and was then weaned to a single therapy if the patient showed improvement. The acid suppression therapy was maintained for at least 3 months, and weaning was not advised until safe feedings were well tolerated. The PPI used was omeprazole (1 mg/kg/day). 3

### Need for Tracheostomy

Tracheostomy was reserved for a restricted group of patients, such as those with multiple comorbidities, multilevel airway obstruction not solved by surgical intervention at this age, or when there were perioperative adverse events requiring tracheostomy. 15

### Statistical Analysis

Data were analyzed using the IBM SPSS Statistics for Windows (IBM Corp., Armonk, NY, United States) software, version 24.0. The normality of the data was tested with the Shapiro-Wilk test. The qualitative data were expressed as numbers and percentages. The associations involving the categorical variables were tested using the Monte Carlo test. The continuous variables were expressed as mean ± standard deviation values. The results were considered significant when *p* ≤ 0.05.

## Results

Among the 123 patients evaluated, 11 were excluded and 112 were included; they had an average age of 4.3 ± 2.2 months at the time of the initial presentation. In total, 5 of them were premature infants, and their gestational age was adjusted. On the initial examination, 61 (54.5%) patients presented type-II laryngomalacia (short aryepiglottic fold with curled epiglottis), 21 (18.75%), type-I laryngomalacia (redundant arytenoid mucosa), 6 (5.4%), type-III laryngomalacia (epiglottic collapse), and 24 (21.43%) patients had mixed type-I and -II laryngomalacia.

A total of 30 cases were diagnosed and presented with associated congenital anomalies; 3 of them had more than 1 anomaly. These anomalies were distributed as follows: 3 cases had Down syndrome, 14 patients had neurological comorbidities, including cerebral palsy (5 cases), epilepsy (6 cases), and febrile convulsions (3 cases), and 13 patients presented cardiac anomalies, including ventricular septal defect (5 cases), persistent ductus arteriosus (2 cases), atrial septal defect (3 cases), tetralogy of Fallot (2 cases), and coarctation of the aorta (1 case). The classification of the patients using the history and symptom score yielded 3 groups: mild – 44 (39.3%); moderate – 48 (42.86%); and severe – 20 (17.85%) ( Table 2 ).

**Table 2:** Application of the history and symptom score to the laryngomalacia patients

	Mild: n (%)	Moderate: n (%)	Severe: n (%)	Total: n (%)
**Inspiratory stridor**				
0	12 (10.71%)	5 (4.46%)	0 (0%)	17 (15.17%)
1	32 (28.6%)	26 (23.21%)	3 (2.7%)	61 (54.5%)
2	0 (0%)	17 (15.17%)	17 (15.17%)	34 (30.6%)
**Cyanotic spells**				
0	44 (39.3%)	44 (39.3%)	6 (5.4%)	94 (83.9%)
3	0 (0%)	4 (3.6%)	14 (12.5%)	18 (16.1%)
**Interrupted sleep**				
0	30 (26.8%)	12 (10.7%)	0 (0%)	42 (37.5%)
1	14 (12.5%)	30 (26.8%)	6 (5.4%)	50 (44.64%)
2	0 (0%)	6 (5.4%)	14 (12.5%)	20 (17.85%)
**Baby growing**				
0	32 (28.6%)	10 (8.9%)	0 (0%)	42 (37.5%)
1	12 (10.7%)	26 (23.21%)	5 (4.46%)	43 (38.4%)
2	0 (0%)	12 (10.71%)	15 (13.4%)	27 (24.1%)
**Choking**				
0	28 (25%)	2 (1.8%)	0 (0%)	30 (26.8%)
1	16 (14.28%)	42 (37.5%)	6 (5.4%)	64 (57.14%)
2	0 (0%)	4 (3.6%)	14 (12.5%)	18 (16.1%)
**Regurgitation**				
0	34 (30.36%)	10 (8.9%)	0 (0%)	44 (39.3%)
1	10 (8.9%)	36 (32.14%)	10 (8.9%)	56 (50%)
2	0 (0%)	2 (1.8%)	10 (8.9%)	12 (10.7%)
**Intensive Care Unit**				
0	42 (37.5%)	33 (29.46%)	3 (2.7%)	78 (69.64%)
1	2 (1.8%)	15 (13.4%)	11 (9.82%)	28 (25%)
2	0 (0%)	0 (0%)	6 (5.4%)	6 (5.4%)
**Total**	44 (39.3%)	48 (42.86%)	20 (17.85%)	112 (100%)

### Mild Cases

On the follow-up of the mild group, 39 patients maintained the same history and symptom score, and, in 5 cases, the score increased to values from 4 to 9, so the examination score was applied to these subjects: their scores were lower than 4, so they continued the conservative treatment.

### Moderate Cases

The examination score was applied to the 48 subjects in the moderate group ( Table 3 ), resulting in 3 subgroups: 13 patients with scores < 4; 22 with scores from 4 to 9; and 13 with scores > 9. The first group was conservatively managed and remained controlled until the end of the follow-up period. Patients in the third group were considered supraglottoplasty candidates. The second group was subjected to the third stage of the assessment, with the application of the investigation score, resulting in 2 subgroups with 11 patients each: one with scores ≤ 5 and the other with scores > 5.

**Table 3:** Application of examination score to the moderate and severe laryngomalacia cases

	Moderate: n (%)	Severe: n (%)	Total: n (%)
**Growth chart**			
0	0 (0%)	0 (0%)	0 (0%)
1	19 (27.9%)	1 (1.5%)	20 (29.41%)
2	29 (42.64%)	19 (27.94%)	48 (70.6%)
**Chest retractions**			
0	11 (16.2%)	0 (0%)	11 (16.2%)
1	13 (19.12%)	6 (8.8%)	19 (27.94%)
2	20 (29.41%)	11 (16.2%)	31 (45.6%)
3	4 (5.9%)	3 (4.41%)	7 (10.3%)
**Degree of collapse**			
0	16 (23.5%)	0 (0%)	16 (23.5%)
1	17 (25%)	14 (20.6%)	31 (45.6%)
2	15 (22.1%)	6 (8.8%)	21 (30.9%)
**Swallowing assessment**			
0	5 (7.4%)	0 (0%)	5 (7.4%)
1	16 (23.5%)	2 (2.9%)	18 (26.5%)
2	27 (39.7%)	18 (26.5%)	45 (66.2%)
**Developmental assessment**			
0	11 (16.2%)	0 (0%)	11 (16.2%)
1	29 (42.65%)	11 (16.2%)	40 (58.82%)
2	8 (11.76%)	9 (8.03%)	17 (25%)
**Congenital anomalies**			
0	26 (32.23%)	12 (17.64%)	38 (55.9%)
1	21 (30.9%)	6 (8.8%)	27 (39.7%)
2	1 (1.5%)	2 (2.9%)	3 (4.41%)
**Total**	48 (70.6%)	20 (29.4%)	68 (100 %)

The 11 cases with scores ≤ 5 were followed-up until the end of the study with a steady course, except for 1 patient who presented deteriorating symptoms; their score increased to the severe category, and the patient underwent supraglottoplasty. The 11 cases with scores > 5 underwent supraglottoplasty, but, postoperatively, 1 subject with Down syndrome and macroglossia had a compromised airway requiring tracheostomy.

### Severe Cases

**Fig. 5:**
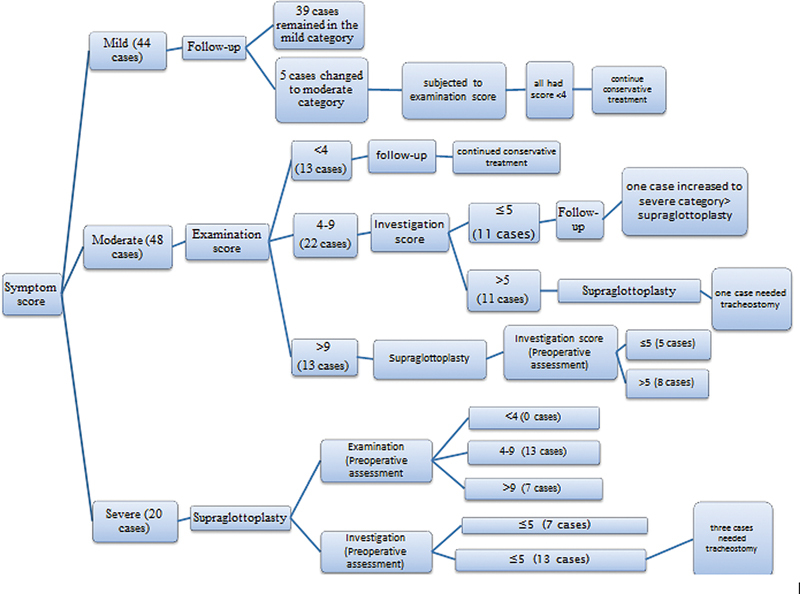
Applied algorithm representing the flow of the patients throughout the study.

All patients in the severe group underwent supraglottoplasty ( Fig. 5 ).

In sum, the examination score was applied to 68 (48 moderate and 20 severe) patients, while the investigation score was applied to 55 (35 moderate and 20 severe) cases ( Table 4 ). All subjects were investigated for GERD though MBS, since the pH meter, although more accurate, was reserved for infants who worsened after the maximum dose of the acid suppression therapy, and for those who presented refractory symptoms following supraglottoplasty or supraglottoplasty failures. 16

Regarding the documented synchronous airway lesions, 15 cases had 1 associated airway lesion, while 11 subjects suffered from 2 concomitant lesions. Those lesions included: tracheomalacia alone, in 8 cases, and combined with bronchomalacia, in 7 cases. Two subjects were diagnosed with vascular rings, and 4 patients presented subglottic stenosis (SGS) alone, 3 of grade I and 1 of grade II, and combined with vocal cord paralysis in 2 cases (grade II). One patient presented a tracheoesophageal fistula, and 2, vallecular cysts with macroglossia. Subjects with tracheomalacia and bronchomalacia were operated on for supraglottoplasty and followed up. Cases of grades I and II underwent balloon dilatation of the subglottic area in addition to supraglottoplasty. Subjects with vallecular cysts and macroglossia underwent cyst removal with coblation midline glossectomy, with an uneventful recovery. The two cases with SGS and bilateral vocal cord paralysis were submitted to tracheostomy in addition to supraglottoplasty. Repair of the tracheoesophageal fistula in the remaining case was performed through supraglottoplasty and tracheostomy. This resulted in a total of 4 tracheostomized infants in the present study. The follow-up period for all cases was of 6 months.

## Discussion

Despite varying presentations of congenital laryngomalacia, most cases are usually managed conservatively until the resolution of the symptoms. 4 The duration of the conservative management, the follow-up intervals, and the surgical decisions show great variability among different institutions, especially in moderately severe and severe cases. In many situations, these aspects depend on the comfort zone of the treating surgeon, the parents' understanding of the situation, and the availability of qualified institutions that can manage urgent airway crises efficiently. A multidisciplinary team consisting of an airway surgeon, a neonatologist, a phoniatrician, and a pediatric anesthetist should be present to establish the appropriate case-specific treatment strategy. The scoring system herein proposed is regarded as a way to aid in the assessment of laryngomalacia cases and stratify them numerically to facilitate the surgeon's decision.

The most common symptom of laryngomalacia, inspiratory stridor is the first symptom assessed in the proposed history and symptom score, with grades similar to those considered previously in pediatric stridor patients. 5 17 18 Feeding problems are the second most common manifestation of this disease, and it is caused by the laryngeal hypotonia present in most cases of laryngomalacia, with subsequent incoordination of sucking, swallowing, and breathing. 4 19 Moreover, gastric distension and vagal stimulation from continuous airway obstruction and limited air entry during feeding lead to regurgitation and/or vomiting. 4 Our scoring system includes an evaluation of these feeding problems based on the symptoms reported by the child's parent/caregiver during and after feeding. Persistent and severe feeding symptoms, as well as choking and regurgitation, were reported to be frequent in infants with severe cases of the disease; 20 21 thus, these patients had higher scores.

There is a general agreement among surgeons 1 regarding the signs of severity of laryngomalacia, which include the presence of severe chest retractions that may result in the development of pectus excavatum, pulmonary hypertension, and cor pulmonale together with severe desaturation during sleep; these signs of severity were assigned values of 3 points in the proposed score. An anatomical description of laryngomalacia has also been suggested as a criterion to select patients for surgery. The severity of the supraglottic collapse is what really matters, regardless of the type of laryngomalacia. 22 Previous studies 22 23 have not found a correlation between morphological type and severity, and Zalzal et al. 24 have recommended surgery for patients whose vocal folds cannot be observed due to supraglottic prolapse, regardless of the type. Thus, the morphological type was not included in the examination score herein proposed, but a degree of invisibility of the laryngeal inlet was considered.

Assessment of swallowing by FEES is a cornerstone in the examination score, as any interruption of the normal pattern is more likely to occur in infants with severe disease. This is supported by Thompson, 4 who reported that infants with severe degrees of airway obstruction often demonstrate difficulty in coordinating sucking, swallowing, and breathing. The FEES examination is the most commonly used and validated instrumental assessment of infant swallowing, although it requires specialized equipment and significant expertise and training in the procedural technique and interpretation of findings. it has also proven to be an appropriate and safe modality to provide a dynamic three-dimensional view of the pharyngeal and laryngeal anatomy that can be helpful in the diagnosis of both airway and swallowing pathologies. 25

The GMDS-III is an instrument to assess mental development in five areas: fine motor skills, gross motor skills, language, and cognitive and personal-social-emotional aspects of infants and children. 9 It is applied since the first month of life, and low scores are more common among infants with severe disease. A developmental score is a reflection of an underdeveloped central nervous system (CNS) and of developmental delay. The correlation between low postnatal scores, stridor, and the development of compromised respiratory function is recognized as an influential factor in the disease's etiology and severity. 26 27

Nearly 25% of laryngomalacia cases present with an associated comorbidity. 4 19 28 29 The severity and duration of laryngomalacia are influenced by the presence of additional congenital anomalies, and worse surgical outcomes have been reported if they are present. So, addressing these co-morbidities and including them in the examination score is extremely important. 28 29 30 Possible associated congenital anomalies are neurologic and congenital heart disease, and they are found more commonly in moderate and severe cases rather than in mild cases. 4 30 This is explained by the fact that congenital anomalies often come in groups with multiple associated comorbidities that augment airway obstruction.

Furthermore, the number of these associated anomalies does matter. Infants with laryngomalacia and an isolated anomaly have been reported 31 to be surgical candidates with a high rate of success in supraglottoplasty. On the other hand, the presence of more than one associated anomaly can adversely affect supraglottoplasty outcomes and may require tracheostomy until resolution or stabilization of the other comorbidities. 32 Infants with 2 medical comorbidities are 5 times more likely to require revision supraglottoplasty, while those with 3 comorbidities are 10 times more likely to require a tracheostomy despite the aggressive management of the disease. 31 Hence, the number of these associated anomalies is highlighted in the new scoring system.

In the new scoring system, the cardiological evaluation should be performed by echocardiography (ECHO) in patients to whom the investigation score will be applied. The coexistence of laryngomalacia and congenital heart disease rarely occurs in mild laryngomalacia cases; it is more frequent in moderate and severe cases, with exacerbation of cyanosis, apnea, and stridor symptoms of laryngomalacia. This association has been reported in 10% of infants with laryngomalacia, 4 33 and up to 34% of them require surgical intervention, 4 34 with higher rates of possible tracheostomy. 31 The degree of heart disease should be evaluated to determine its extent and detect the development of pulmonary hypertension and right-sided heart failure. 1 4 In laryngomalacia cases, the sequelae of a cardiac condition, hypoxia and cyanosis, can be partially improved through supraglottoplasty, keeping in mind the fact that the patient will not completely improve unless the cardiac problem is corrected. It is the duty of the pediatric airway board to anticipate and prevent these sequelae. Assigning these sequelae a high value in the current score helps physicians pay attention to and reduce the incidence of such events.

Neurologic disease is the second most commonly reported medical comorbidity, with an incidence of 8 to 45%. 4 Neurologic affection is thought to influence vagal nerve function of laryngeal tone at the central level, thereby contributing to symptom severity. Infants with neurologic disease require surgical intervention at higher rates than those without it, and up to 60% may require tracheostomy. 16 Nevertheless, the outcome of supraglottoplasty among the patients with neurologic disease in the present study was successful, with no additional tracheostomy required.

The relationship between obstructive sleep apnea (OSA) and laryngomalacia has been an area of significant investigation for several years. 5 The occurrence of OSA is associated with polysomnographic evidence of nighttime airway obstruction and desaturation, and this has been reported 5 to be proportionate to the degree of severity. In the clinical practice, it is difficult to use polysomnography despite the recommendation for its routine use. Though less specific, overnight pulse oximetry is more available, and studies have reported 35 36 37 that it facilitates clinical decision-making. Although not equivalent to the gold standard, which is polysomnography, it was applied to all subjects in the present study, and it enabled direct comparisons and staging of the patients.

Synchronous airway lesions (SALs) are commonly present in cases of laryngomalacia, with an incidence that ranges from 7.5 to 64%. 6 30 38 It has been demonstrated 30 38 that the association of SAL results in worse obstructive symptoms preoperatively as well as an annoying postoperative course. Moreover, SAL increases the severity of the symptoms through a cumulative effect on airway obstruction, potentiating GERD. 4 30 38 The most commonly associated SALs are subglottic stenosis and tracheomalacia, which are the following findings in the current case series. Dickson et al. 38 mentioned that SAL contributes to the need for surgical intervention even among mild or moderate laryngomalacia cases, with longer hospital stays and raised susceptibility to requiring airway support or reintubation. Thus, scaling of associated SAL is a critical point in the assessment, and the presence of more than one SAL should be assigned a high score.

The most common medical comorbidity is GERD, which is present in 65 to 100% of laryngomalacia cases. 4 19 39 40 Acid regurgitation often results in inflammation and edema of the upper airway, creating a vicious cycle of reflux and airway edema until one or both are alleviated. 40 41 These induced edematous changes are partially responsible for the prolapse of the supra-arytenoid mucosa into the glottic inlet in type-I laryngomalacia. 30 Those effects are prominent in infants with moderate to severe laryngomalacia. 42 43 44

In the literature, 1 45 46 most cases of laryngomalacia are managed conservatively, while up to 15 to 25% require surgery. In comparison to the present study, we observed a relatively higher incidence of surgical patients: 45 cases (40.2%). This is explained by the type of flow of cases, as most of the mild cases could go mis- or undiagnosed or treated conservatively by pediatricians. Of the 45 cases found in the present study, 20 were considered severe, 13 were moderate cases with high examination scores, 11 were moderate cases with examination scores from 4 to 9, but with high investigation scores, and there was a single case with an examination score from 4 to 9, with a low investigation score. It is notable that none of the cases considered mild according to the history and symptom score required surgery during the follow-up period. Even the 5 cases with increased scores during the follow-up remained within the conservative category.

Shivnani et al. 47 proposed a scoring system based on the clinical profile staging and management of infants/children diagnosed with laryngomalacia. Their score was based on a retrospective observational study, and patients with acquired laryngomalacia associated with a congenital cardiac anomaly, neurological disorders, or synchronous lesions of the larynx were excluded from their work. They also recommended the validation of their scoring system through multicentric prospective studies.

A numerical score makes disease grading simpler, aiding physicians in their practice. Moreover, the novel score herein proposed can serve as a universal language to expressing grade of laryngomalacia both subjectively and objectively and provide a base for a simplified stepwise approach to the management of different cases of the disease.

The main limitation of the current study is the difficulty in establishing a strict cut-off point for the three grades of laryngomalacia and deciding to proceed with the conservative or surgical managements based on a numerical value. Taking into consideration certain variables, such as the parents' apprehension, level of schooling, and social background, some malleability is needed, especially with borderline cases. Therefore, the score should be a complementary tool to the opinion of the airway board. The history and symptom score is subjective. Due to the hazards of right cardiac catheterization and the young age of our patients, we measured pulmonary hypertension using transthoracic ECHO instead.

## Conclusion

The scoring system herein proposed is a reasonable and applicable way to standardize our practice regarding supraglottoplasty. We have used it at our institution to provide a quantitative measure of the extent of laryngomalacia and its proper management. The proposed score and the stepwise algorithm are easy to learn and apply for general practitioners, otorhinolaryngologists, and pediatricians, and it aids in the widespread improvement in laryngomalacia management.

**Table 4:** Application of investigation the score to the moderate and severe laryngomalacia cases

	Moderate: n (%)	Severe: n (%)	Total: n (%)
**Echocardiography**			
0	5 (9.1%)	8 (14.5%)	13 (23.6%)
1	10 (18.2%)	3 (5.5%)	13 (23.6%)
2	18 (32.73%)	6 (10.9%)	24 (43.6%)
3	2 (3.6%)	3 (5.5%)	5 (9.1%)
**Overnight pulse oximetry**			
0	1 (1.8%)	0 (0%)	1 (1.8%)
1	10 (18.2%)	0 (0%)	10 (18.2%)
2	21 (38.2%)	14 (25.5%)	35 (63.6%)
3	3 (5.5%)	6 (10.9%)	9 (16.4%)
**Synchronous airway lesions**			
0	21 (38.2%)	8 (14.5%)	29 (52.7%)
1	10 (18.2%)	5 (9.1%)	15 (27.3%)
2	4 (7.3%)	7 (12.7%)	11 (20%)
**Assessment of gastroesophageal reflux disease**			
0	6 (10.9%)	0 (0%)	6 (10.9%)
1	11 (20%)	6 (10.9%)	17 (30.9%)
2	18 (32.7%)	14 (25.5%)	32 (58.1%)
**Total**	35 (63.6%)	20 (36.4%)	55 (100%)
